# The effectiveness of negative-pressure wound therapy for wound healing after stoma reversal: a randomised control study (SR-PICO study)

**DOI:** 10.1186/s13063-019-3925-z

**Published:** 2020-01-06

**Authors:** Sohyun Kim, Sung Il Kang

**Affiliations:** Department of Surgery, Yeungnam University Medical Center, Yeungnam University College of Medicine, 170 Hyeonchung-ro, Nam-gu, Daegu, 42415 South Korea

**Keywords:** Negative-pressure wound therapy, PICO™ system, Purse-string suture, Stoma reversal

## Abstract

**Background:**

Although the wound-healing period for purse-string closure (PSC) after stoma reversal is longer than that required for the primary closure method, the rate of wound infection is reduced. The application of negative-pressure wound therapy (NPWT) can reduce the healing period for many types of wounds. Herein, we describe a planned trial to test the hypothesis that NPWT can reduce the healing period for PSC after stoma reversal.

**Methods/design:**

Patients undergoing stoma reversal will be recruited and allocated into intervention and control groups, with 1:1 randomisation. Patients in the control group will receive standard postsurgical wound care; patients in the intervention group will receive NPWT using the PICO™ system. The target sample size will be 38 patients, as this will provide 80% power at the 5% level of significance to detect a 7-day reduction in the wound-healing period in the intervention group compared to that in the control group. The primary endpoint will be the duration to wound healing, defined as the time to nearly complete epithelisation of the wound, without any discharge or surgical site infection (SSI). Secondary endpoints will be the SSI rate, length of postoperative hospital stay, number of wound dressings and visits to the hospital for wound dressing after discharge, total cost of wound dressings, and patient and observer scar assessment scale scores.

**Discussion:**

The results of this planned randomised controlled study will clarify the role of NPWT in patients undergoing stoma reversal and strengthen the rationale for choosing a dressing technique.

**Trial registration:**

Clinical Research Information Service (CRIS), KCT0004063. Registered on 6 June 2019.

## Background

Surgeons form temporary stomas to prevent disastrous complications due to anastomotic leakage after colorectal resection, or to maintain bowel continuity in challenging cases of emergency bowel surgery. Patients with a temporary stoma generally undergo reversal surgery a few weeks to several months later. However, stoma reversal surgery has various complications, with the most common being surgical site infection (SSI) [1]. Stoma site closure is generally classified as a class 3, clean-contaminated wound, which has a high risk of SSI [2, 3]. Indeed, the incidence of SSI in cases of primary closure of the skin after stoma reversal is as high as 40% [4, 5].

**Fig. 1:**
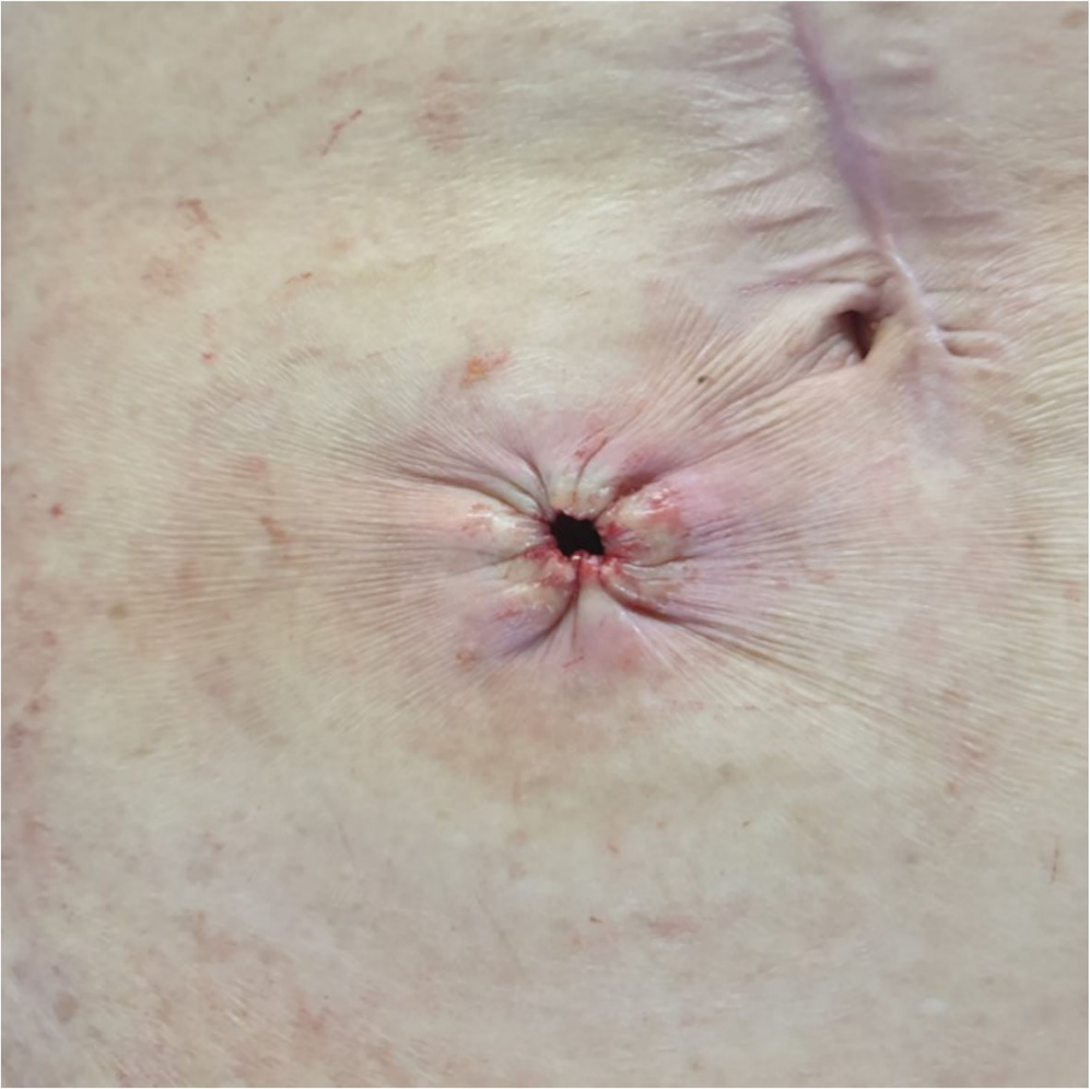
Purse-string suture after stoma reversal

The purse-string closure (PSC) method was developed in an effort to reduce the rate of SSI at the stoma reversal site, and is now widely used for skin closure after stoma reversal surgery (Fig. 1). In two previous randomised control trials (RCTs), the SSI rate was lower in patients who underwent PSC after stoma reversal than in those who underwent conventional linear closure after stoma reversal (0% vs 36.1% and 2% vs 15%, respectively) [6, 7]. Furthermore, a meta-analysis of four RCTs reported significantly fewer instances of SSI with PSC than with conventional primary closure (risk difference, − 0.25; 95% confidence interval, − 0.36 to − 0.15; *p* < 0.00001) [8]. Thus, the SSI rate decreases dramatically when PSC is used for skin closure after stoma reversal.

However, a hole 0.5–1 cm in diameter is formed during the PSC method for discharge drainage, and secondary healing of this skin hole is induced. Thus, a disadvantage of PSC is that the wound-healing period is prolonged compared to that for primary closure. Specifically, the mean wound-healing time is 2 weeks in patients who undergo primary closure of the skin and do not develop an SSI, but is 3.8 weeks in patients who undergo PSC after stoma reversal [6].

Negative-pressure wound therapy (NPWT) was first introduced in 1997 [9]. Since then, NPWT has been applied to various types of wounds worldwide, and numerous studies have shown its effectiveness in reducing the SSI rate and promoting wound healing [10–12]. A Cochrane Review published in 2018 specifically noted that the application of an NPWT device reduces the wound-healing period in the treatment of diabetic feet [13]. We hypothesised that the application of NPWT would similarly reduce the wound-healing period, as well as the SSI rate, in patients undergoing PSC after stoma reversal. Therefore, we designed a study, described herein, to evaluate the efficacy of NPWT in reducing the wound-healing period for PSC after stoma reversal.

## Method

### Study design

**Fig. 2:**
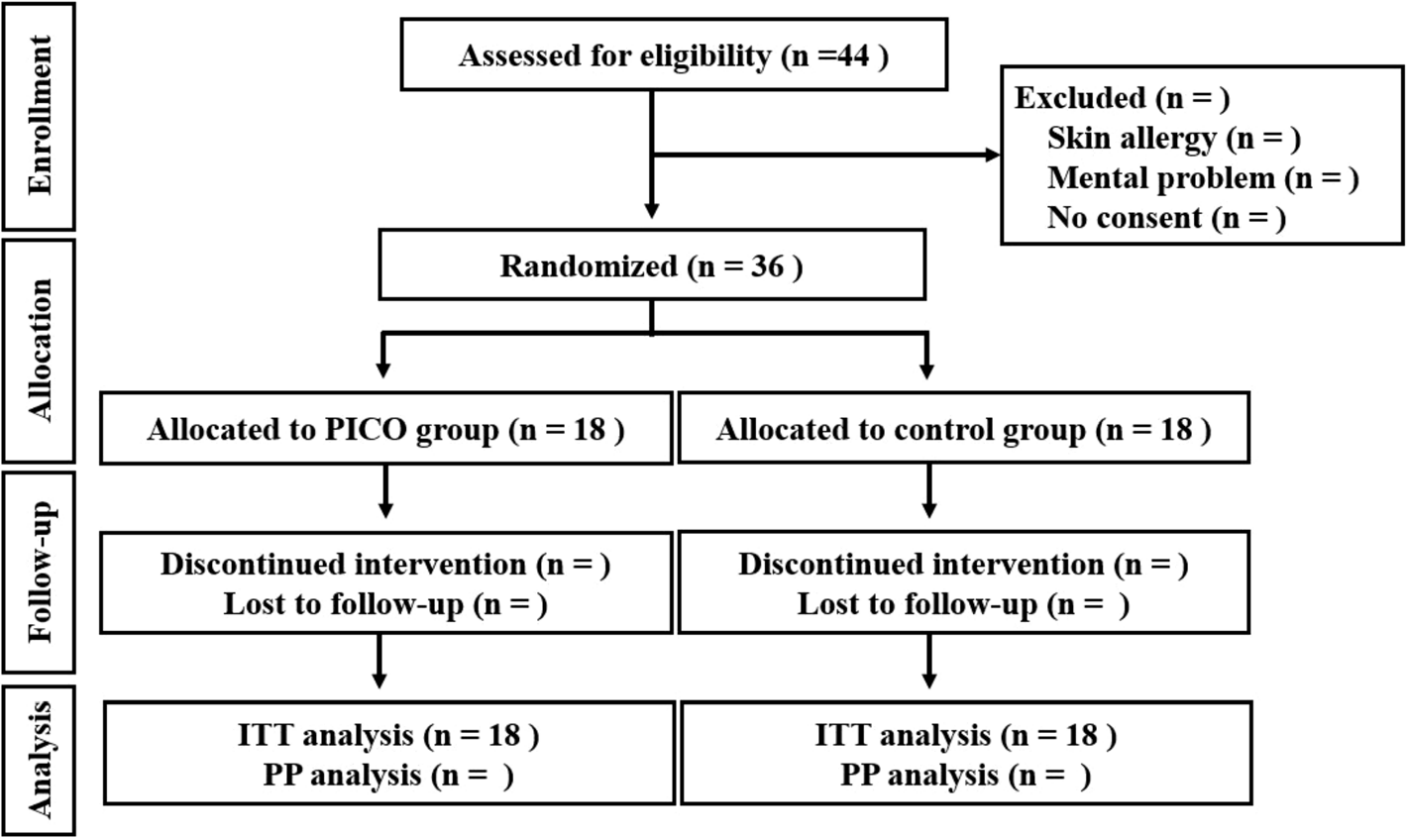
SR-PICO study flowchart. The anticipated number of patients assessed for eligibility is based on the mean number of stoma reversal cases per year (*n* = 22) at our institution. ITT intention to treat, PICO PICO™ system, PP per protocol

A single-centre RCT will be conducted. The wound-healing period with the application of a NPWT device after stoma reversal using the PSC method will compared to that with a conventional dressing. The study design follows the recommendations of the Standard Protocol Items: Recommendations for Interventional Trials (SPIRIT) guidelines. The study flowchart is shown in Fig. 2 Fig. 4 Additional file 1.

### Study population

Patients aged > 20 years who will receive a temporary stoma in a stoma-reversal surgery will be recruited at Yeungnam University Medical Center in South Korea. Patients with a skin disease, including allergies that would interfere with the application or evaluation of the NPWT device, an inability to express himself/herself due to conditions such as dementia and intellectual disabilities, and an inability or unwillingness to provide informed consent will be excluded.

### Endpoints

The primary endpoint will be the duration of wound healing, defined as the time to nearly complete epithelisation of the wound, without any discharge or SSI, which is similar to the definition used in the study by Uchino et al. [3]. The determination of the degree of wound healing will be made by the researcher and a single wound/ostomy-specialised nurse. Another colorectal surgeon (who will be blinded to the patient’s identity and treatment assignment) will also judge the extent of wound healing via photographs of the wound. Wound healing will be considered complete by the consensus of all three reviewers.

Secondary endpoints comprise the SSI rate, length of postoperative hospital stay, number of wound dressings and visits to the hospital for wound dressing after discharge, total cost of wound dressings, and patient and observer scar assessment scale (POSAS) scores [14]. The presence of an SSI will be decided by the researcher and wound/ostomy-specialised nurse, using the Center for Disease Control definition of SSI [15]. The evaluation of SSI will be performed using the same process as that for the assessment of wound healing. The POSAS will be completed at the outpatient clinic approximately 1 month after surgery.

### Interventions

All participants will be randomised to either the intervention group or the control group, using a 1:1 ratio. The randomisation allocation will be generated using a computerised randomisation system and applied immediately after fascia occlusion to avoid a performance bias.

**Fig. 3:**
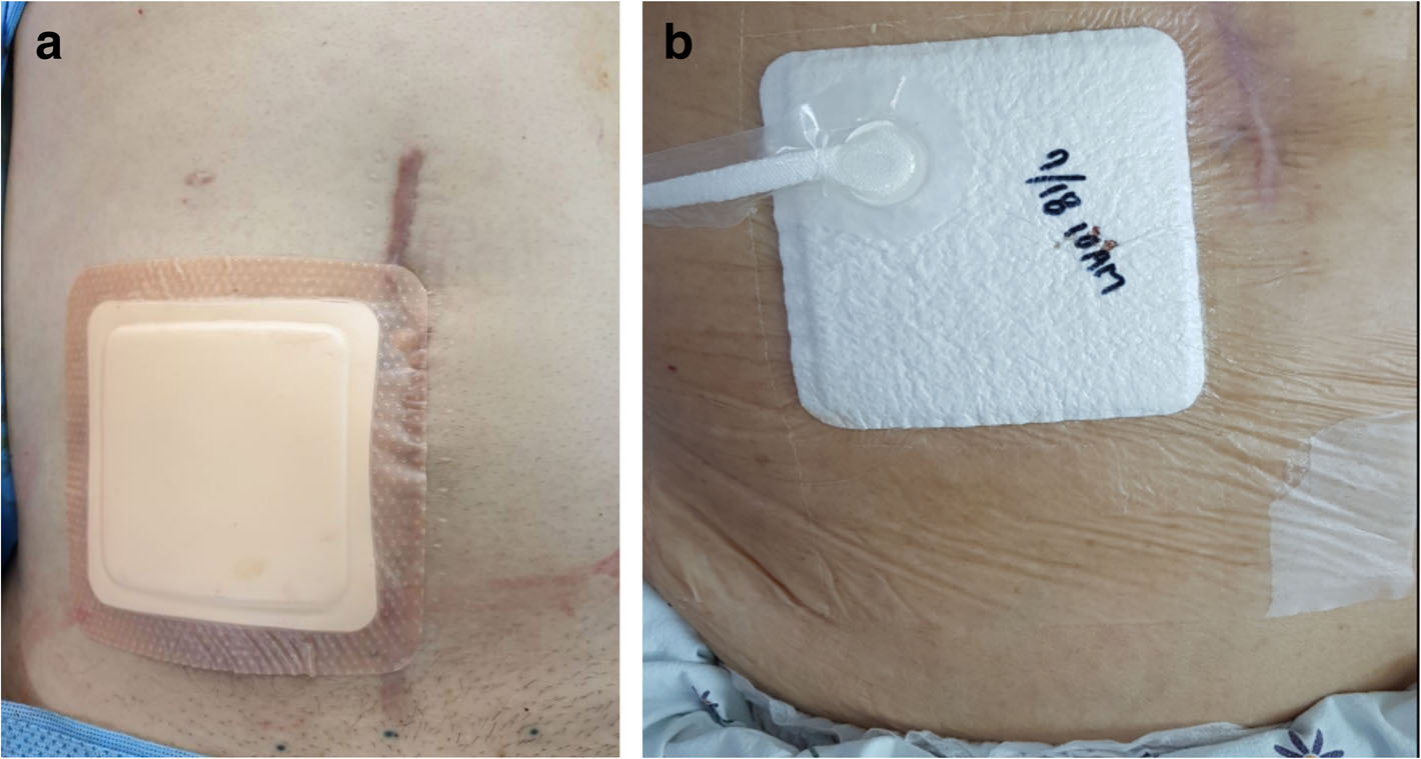
**a** Simple dressing application used in the control group. **b** PICO™ application used in the intervention group

Stoma reversal will be performed using standard techniques, with the patient under either spinal or general endotracheal anaesthesia, and the bowel anastomosis will be either stapled or hand sewn at the discretion of the surgeon. After fascia closure, skin closure will be performed with the PSC method using absorbable monofilament suture material (2–0 Monocryl; Ethicon, Cincinnati, OH, USA), leaving an opening ≤ 0.5 cm (approximately). Subsequently, a simple, transparent, waterproof dressing will be applied to patients in the control group, and a PICO™ portable NPWT device (Smith & Nephew Healthcare, Hull, UK) dressing will be applied to patients in the intervention group (Fig. 3).

**Fig. 4:**
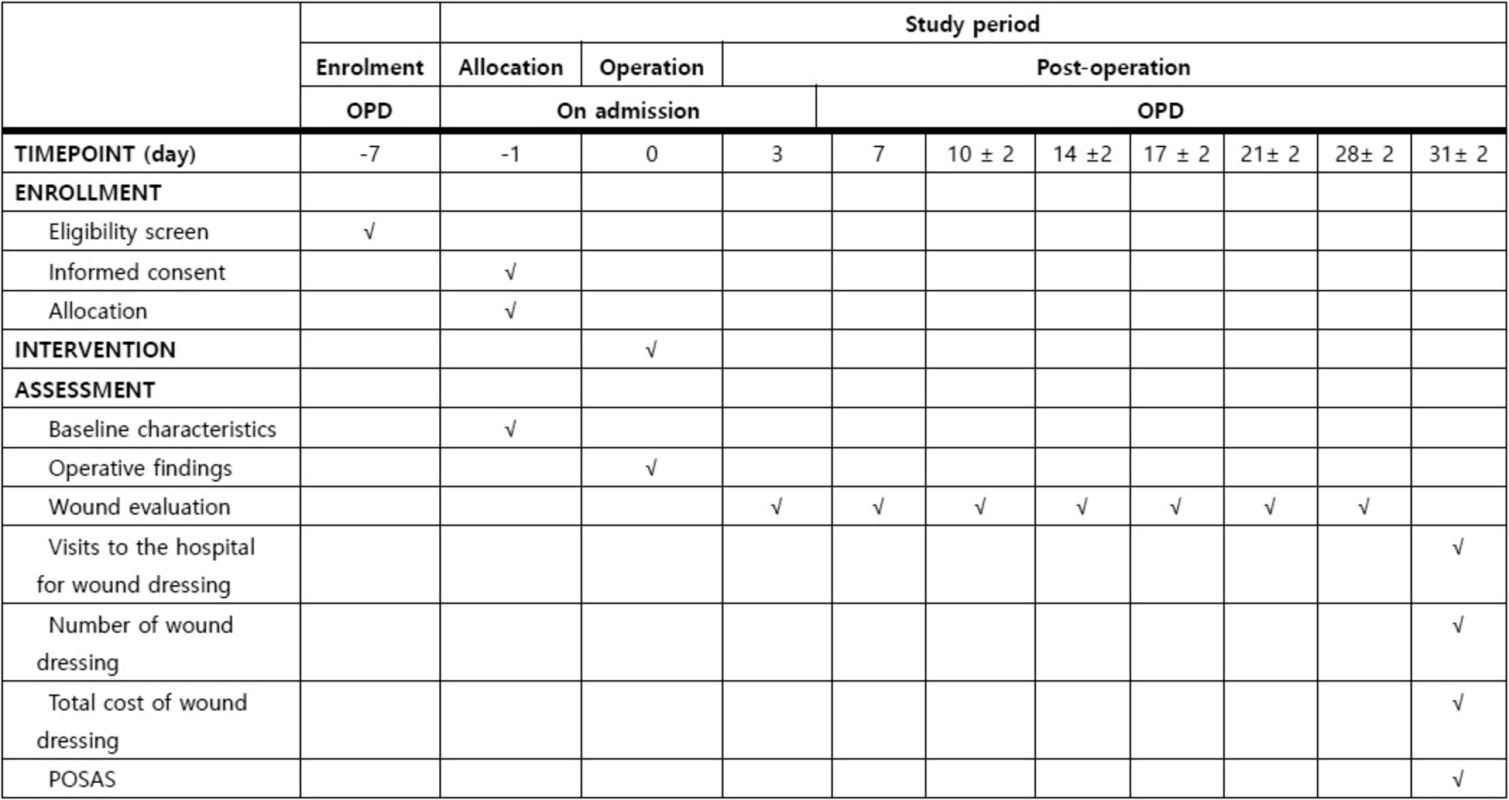
Schedule of assessment

Routine dressing changes will be performed once a day in the control group and twice a week in the intervention group. Additional dressing changes will be performed as needed during the postoperative hospital stay (i.e. when the dressing material is wet with discharge). If patients in the intervention group require dressing changes more than once a day during hospitalisation, we plan to replace the NPWT dressings with conventional dressings due to cost-effectiveness concerns, as NPWT dressing material is far more expensive than is conventional dressing material.

Outpatient follow-up after discharge will be scheduled twice a week until wound healing is judged as complete. Prophylactic antibiotics (second-generation cephalosporin) will be administered 1 h before surgery, and additional antibiotics will not be permitted unless there are signs of infection during the postoperative period.

### Sample size calculation

The target sample size will be 38 participants, as this will provide 80% power at the 5% (two-sided) level of significance to detect a 7-day reduction in the wound-healing period in the intervention group compared to that in the control group (superiority design, 19 ± 7 days vs 26 ± 7 days), with allowance for 10% attrition. The anticipated wound-healing period in the control group is based on data from a previous RCT that compared the healing times of the stoma reversal site between conventional linear closure and PSC [6]. We expect to screen approximately 44 patients for eligibility during the study period (*n* = 22 per year), based on the mean number of stoma reversal cases at our institution in the last 2 years. The length of the study period was decided with consideration of the expected screening number and a dropout rate of 20% (due to exclusion and refusal to consent).

### Data collection and management

All data for this trial will be collected after obtaining informed consent from the participants 1 day before surgery. Paper case report forms will be used to record all baseline demographics and other data for each participant. Serial wound photographs will be taken by the investigators during dressing changes, from the immediate postoperative day to the discharge day during hospital stays, and at every visit to the outpatient clinic. The photographic data will be used to determine the degree of SSI and wound healing. All photographic data will be kept on file in an anonymised fashion, in the same manner as for the patient’s other data. All information acquired in this study will be anonymised through the assignment of a trial identification number, which will be used only for this trial and its research purposes, and will only be accessible to authorised persons. All data will be stored for 3 years from study completion.

All collected data and the participant’s safety will be monitored by a data and safety monitoring committee. The committee will meet once a year to monitor the study. Because of the proven safety of NPWT, the study is expected to be safe for the participants. Nevertheless, the investigators will monitor the participants for any severe adverse events related to the study treatment (e.g. a severe skin allergy due to the dressing materials) and will immediately report these to the data and safety monitoring committee. The investigators will change the study protocol according to the recommendations of the committee.

### Statistical analysis

No interim analyses will be conducted due to the small sample size of the study. All analyses of the primary/secondary endpoints will be conducted with the intention-to-treat population. A per-protocol analysis will be also performed for further comparisons. Normally distributed data will be examined using Student’s *t* test. Non-normally distributed data will be examined using the Mann–Whitney *U* test. The chi-square or Fisher’s exact test will be used to examine categorical variables. *p* < 0.05 will be considered statistically significant.

### Ethics

This study will be conducted in accordance with the ethical standards of the Declaration of Helsinki. This trial was approved by the institutional review board at Yeungnam University Medical Center on 12 June 2019 (IRB No. 2019–04–031-002) and registered with the clinical research information service (CRIS KCT0004063). The researcher will obtain written informed consent from the participants. The results of this study will be published in peer-reviewed medical journals.

## Discussion

The primary objective of this RCT will be to evaluate the application of NPWT to PSC after stoma reversal regarding its effectiveness in reducing the wound-healing period.

Several mechanisms contribute to the efficacy of NPWT. NPWT reduces seroma formation in a wound and stimulates angiogenesis and granulation. Negative pressure also creates a hypoxic environment in a wound, resulting in the upregulation of inflammatory cytokines, which stimulates wound healing [10, 16].

One previous RCT evaluated the application of NPWT to laparotomy wounds following abdominal surgery [10]. In this previous study, the prophylactic use of NPWT reduced the SSI rate compared to that in the control group. However, the study did not compare the wound-healing period between the two groups. Two additional studies evaluated the application of NPWT to ileostomy closure sites and demonstrated the efficacy of NPWT in reducing the SSI rate [17, 18]. However, these previous studies focused on patients with primary linear closure of stoma wounds and did not evaluate the wound-healing period.

Uchino et al. [3] conducted an RCT with a similar primary endpoint to that in the present planned trial. The authors failed to demonstrate the efficacy of NPWT in significantly reducing the wound-healing period. However, the participants of this previous RCT comprised patients with ulcerative colitis, and the opening of the PSC was wider (8 mm) than that in the present planned trial (< 5 mm). As the opening of the PSC in the present planned trial will be ≤ 0.5 cm, we believe that our trial will more likely demonstrate positive results, which may be generalised to clinical practice. Another strength of our planned trial is that we will evaluate the cost of the wound dressings, number of wound dressings, and number of hospital visits for wound dressings after discharge, as direct and indirect assessments of the convenience level and medical cost.

Although we designed this trial as a prospective RCT, several limitations exist. First, this will be a single-centre study, with a small sample size. However, we believe that the study will be adequately powered to evaluate the primary endpoint. Additionally, single-centre studies have advantages with respect to data collection consistency. Second, the assessment of the wound-healing period as a primary endpoint is subjective in nature. We plan to overcome this issue by utilising three experts to evaluate the wound directly or via photographs of the wound. However, two of the wound reviewers cannot be blinded, as one is the operator and the other is the nurse who will dress the wounds. Only one surgeon, who will evaluate the wound based on photographs, can be blinded. Third, we will enrol all types of stoma reversal surgeries, regardless of the patient’s stoma type (e.g. loop ileostomy, loop colostomy, end colostomy, etc.). This may lead to a large bias in the study; however, we believe that the inclusion of all stoma types will benefit the generalisability of the results. The stoma reversal technique will be performed in the same manner regardless of the stoma type, and the endpoints of the SR-PICO study will be limited to the evaluation of the wound at the stoma reversal site.

In conclusion, the SR-PICO trial is a planned RCT to evaluate the efficacy of NPWT in reducing the wound-healing period in patients who undergo stoma closure using the PSC method. We believe that the results of the SR-PICO trial will clarify the role of NPWT in patients undergoing stoma reversal and strengthen the rationale for choosing a dressing technique.

### Trial status

This trial (protocol version 1.1) is in the ongoing recruitment phase, which started on 25 June 2019. The first participant was enrolled on 14 July 2019 and seven participants have been enrolled in the study as of 22 October 2019. The study will be completed on 30 June 2021.

## Supplementary information

**Additional file 1.** SPIRIT 2013 Checklist: Recommended items to address in a clinical trial protocol and related documents.

## Data Availability

The data collected in the SR-PICO study will be publicly available online upon trial completion.

## Abbreviations

CRIS: Clinical Research Information Service
NPWT: Negative-pressure wound therapy
POSAS: Patient and observer scar assessment scale
PSC: Purse-string closure
RCT: Randomised controlled trial
SSI: Surgical site infection
